# Unplanned Healthcare Encounters in Drug-Resistant Urinary Tract Infections in Emergency Departments

**DOI:** 10.7759/cureus.85138

**Published:** 2025-05-31

**Authors:** Jessica M Brochu, Satheesh Gunaga, Rachel M Kenney, Michael P Veve

**Affiliations:** 1 Critical Care and Pharmacy, Henry Ford Health System, Detroit, USA; 2 Emergency Medicine, Henry Ford Wyandotte Hospital and Envision Healthcare, Wyandotte, USA; 3 Osteopathic Medical Specialties, Michigan State University College of Osteopathic Medicine, East Lansing, USA; 4 Infectious Disease and Pharmacy, Henry Ford Health System, Detroit, USA; 5 Pharmacy Practice, Eugene Applebaum College of Pharmacy and Health Sciences, Wayne State University, Detroit, USA

**Keywords:** antibiotic resistance, antimicrobial stewardship, emergency department, esbl, urinary tract infection

## Abstract

Introduction

The treatment of extended-spectrum β-lactamase (ESBL)-producing urinary tract infections (UTIs) in the emergency department (ED) is challenging due to the limited oral treatment options available. The purpose of this study was to describe the treatment and outcomes of ESBL UTIs in the ED and to determine risk factors associated with secondary UTI-related unplanned healthcare encounters.

Methods

This was an institutional review board (IRB)-approved, retrospective cohort study of patients discharged from the ED with an ESBL UTI. The primary outcome was any UTI-related unplanned healthcare encounter within 30 days of the index ED visit. Unplanned healthcare encounters included phone/virtual visits, clinic visits, ED visits, and hospitalizations. Patients of ≥18 years of age treated for symptomatic UTI were included. Logistic regression was used to identify exposures independently associated with UTI-related unplanned healthcare encounters.

Results

A total of 162 patients were included, of which 103 (64%) experienced an unplanned healthcare encounter. The most common UTIs were complicated lower (71, 44%), complicated upper (57, 35%), catheter-related (24, 15%), and uncomplicated cystitis (10, 6%). Nitrofurantoin demonstrated to have in vitro activity in 121 (75%) patients, aminoglycosides in 117 (72%) patients, trimethoprim/sulfamethoxazole (TMP/SMX) in 66 (41%) patients, and fluoroquinolones in 62 (38%) patients. Of the 103 patients who experienced an unplanned healthcare encounter, 76 (74%) received inactive empiric antibiotic treatment. Oral β-lactams were most commonly prescribed, accounting for 66 (41%) of all initial prescriptions. Of the 81 patients with lower UTI, only 20 (25%) received a prescription for nitrofurantoin. Factors associated with UTI-related unplanned healthcare encounters included chronic kidney disease (CKD) (adjusted odds ratio {adjOR}, 3.4; 95% confidence interval {CI}, 1.2-9.5) and empiric oral β-lactam use (adjOR, 3.2; 95% CI, 1.5-6.6).

Conclusions

Patients with CKD or who received empiric oral β-lactam treatment more commonly experienced an ESBL UTI-related unplanned healthcare encounter. Prescribing first-line therapy with nitrofurantoin for lower UTI is a potential area for improvement.

## Introduction

Urinary tract infections (UTIs) caused by extended-spectrum β-lactamase (ESBL)-producing Enterobacterales have emerged as a significant global health concern [1]. ESBLs are enzymes produced by gram-negative bacteria that confer resistance to penicillins, monobactams, and oxyimino-cephalosporins. However, plasmids that encode for ESBL enzymes often confer resistance to other antibiotic classes as well, including oral agents, which leaves intravenous (IV) carbapenems as preferred therapy [2]. The management of ESBL UTIs in the emergency department (ED) is particularly challenging due to diagnostic challenges and limited oral treatment options available in the outpatient setting [3,4]. Despite the expanding knowledge and surveillance of ESBL infections in various healthcare settings, there remains a crucial need for further investigation of their specific implications in acute care settings, especially within the ED [5-7].

Rapid diagnostic tests for ESBL UTIs are not readily accessible, and despite some modest information about ESBL UTI risk factors, more data are needed, and the proper utilization of risk factor scoring tools is challenging at the bedside [8,9]. The available literature on ESBL UTIs in the ED is still in its early stages but is continuously expanding. Primarily composed of retrospective studies and epidemiological surveillance research, these studies have provided valuable initial insights into the prevalence and characteristics of ESBL UTIs in the ED [10,11]. A series of recent research has identified several predictors of ESBL UTIs in ED patients, such as prior IV antibiotic use, recent surgical procedures, persistent UTI despite antibiotic therapy, and various demographic and medical history-related risk factors [8,9].

Despite the growing body of literature, there are still significant gaps in knowledge, particularly regarding the implications of the management of UTIs caused by ESBL-producing Enterobacterales treated in the ED. A better understanding of the impact of ED prescribing patterns and patient-specific characteristics on outcomes is necessary to develop effective antimicrobial stewardship strategies for ESBL UTIs. The objective of this study was to provide a comprehensive description of the treatment and outcomes of patients with ESBL UTIs treated in the ED to identify opportunities for optimizing ED-targeted antimicrobial stewardship efforts and inform best practices.

## Materials and methods

This institutional review board (IRB)-approved, retrospective cohort study was conducted at six EDs within an integrated metropolitan health system that sees approximately 350,000 ED visits each year. The six distinct EDs include a large urban tertiary ED, four community-based EDs, and one freestanding ED. These sites were strategically chosen to provide a diverse representation of patient populations in our sample. Over the course of two years, approximately 33,000 ED patients were diagnosed with a UTI at one of the participating study sites. Of these, 809 patients were discharged home from the ED with an ESBL-producing pathogen isolated from their urine culture, representing 2.45% of total UTI cases.

Patients of ≥18 years old presenting to and discharged from an ED with UTI diagnostic criteria between January 2020 and November 2022 were included. To address the potential for asymptomatic bacteriuria, patients who presented to an ED with altered mental status as their only UTI symptom were excluded. Patients with a history of renal transplant, abnormal urinary tract anatomy, a creatinine clearance of <30 mL/minute, and expected or confirmed prostatitis or those currently taking active antimicrobial therapy were excluded to prevent including patients with more complex disease that could impact the primary study endpoint. Patient cohorts were defined by those who did and did not have any subsequent UTI-related unplanned healthcare encounter within 30 days of their initial ED visit.

UTI was defined according to classification, being either uncomplicated cystitis, complicated cystitis, complicated upper urinary tract infection, or catheter-associated. These classifications were made according to the definitions in Table 1, as defined by the Michigan Hospital Medicine Safety Consortium [12].

**Table 1:** Definitions used for urinary tract infection classification. UTI, urinary tract infection; ESBL, extended-spectrum β-lactamase; CFU, colony-forming units

UTI Type	Definition
Uncomplicated cystitis (lower tract infection)	Symptomatic plus ESBL growth of ≥105 CFU/mL in immunocompetent, pre-menopausal, nonpregnant women with a normal urinary tract without obstruction, urinary catheters, or symptoms of upper tract infection (flank pain). Symptomatic is defined as at least one of the following with an acute onset: dysuria, frequency, urgency, suprapubic pain/tenderness, incontinence, or gross hematuria.
Complicated cystitis (lower tract infection)	Symptoms plus ESBL growth of ≥105 CFU/mL in patients without symptoms of upper tract infection (flank pain) who do not meet the criteria for uncomplicated cystitis (i.e., male patients, immunosuppressed patients, and patients with urinary catheters). Symptoms are defined as at least one of the following with an acute onset: dysuria, frequency, urgency, suprapubic pain/tenderness, incontinence, or gross hematuria.
Complicated upper urinary tract infection	ESBL growth of ≥105 CFU/mL plus symptoms of upper tract infection or pyelonephritis. Symptoms include costovertebral tenderness, flank pain, and/or fever or other evidence of urinary infection involving the ureters or kidneys.
Catheter-associated	ESBL growth of ≥102 CFU/mL from a single catheter urine specimen plus urinary symptoms. Symptoms are defined as at least one of the following with an acute onset: dysuria, frequency, urgency, suprapubic pain/tenderness, incontinence, gross hematuria, or spasticity/autonomic hyperreflexia in a spinal cord injury patient.

Patients were required to have a urine culture obtained at the initial ED visit, which resulted in a single species of ESBL-producing gram-negative Enterobacterales confirmed through in vitro susceptibility testing. Susceptibility testing was performed by a centralized clinical microbiology laboratory using Vitek® 2 (bioMérieux, Marcy-l’Étoile, France). Isolates were reported as ESBL-producing if the cefazolin minimum inhibitory concentration (MIC) was ≥8 mcg/mL, ceftriaxone MIC ≥2 mcg/mL, and aztreonam MIC ≥2 mcg/mL and the ESBL test was positive on automated susceptibility testing.

The primary outcome was any UTI-related unplanned healthcare encounter within 30 days of the initial ED visit. Unplanned healthcare encounters included all phone/virtual visits, ED visits, clinic visits, and/or hospitalizations due to ongoing or worsening UTI. This definition of unplanned healthcare encounter was chosen to capture any additional encounter(s) patients had with the healthcare system regarding the UTI initially addressed at the index ED visit. Phone/virtual visits included all outreach attempts made by the ED team in response to the resulting culture data post ED discharge. Secondary endpoints included urine culture data such as resulting pathogen and susceptibility patterns, along with initial and definite antimicrobial therapy, which was further classified as active or not active determined by in vitro pathogen susceptibilities.

Patient data were identified and extracted from the electronic medical record using Microsoft SQL Server (Microsoft Corp., Redmond, WA). An SQL query of patients with an ESBL gram-negative organism identified from a urine culture was used as a basis for patient screening. Subsequently, patients were manually screened for inclusion using the criteria above. Data were then collected using a standardized, electronic case report form. A calculated sample size of 162 patients would provide 80% power to detect a 60% incidence of treatment failure among our population. The incidence of treatment failure was estimated using published literature, reporting that 61.4% of patients with a UTI caused by a multidrug-resistant pathogen would experience treatment failure following initial therapy [13]. Patients were randomized and screened for inclusion/exclusion criteria until the sample size was met.

Categorical data were described using frequencies and percentages; continuous data were described using medians and interquartile range (IQR). Bivariate analyses were used to compare patients who did and did not experience a UTI-related unplanned healthcare encounter. Comparisons were made using the chi-square test or Fisher’s exact test for nominal data, whereas the Mann-Whitney U-test was used to compare continuous variables. A p-value of less than 0.05 was considered statistically significant. In order to determine which patients are most likely to experience a UTI-related unplanned healthcare encounter, multivariable logistic regression was used to identify exposures independently associated with the outcome while adjusting for confounders. Variables found to have an association with UTI-related unplanned healthcare encounter from bivariate analysis (P<0.2) were entered into a backward, stepwise logistic regression model using an n-to-K ratio of 10:1. Variables were explored for covariability prior to inclusion into the logistic regression model. All statistics were performed using SPSS Statistics for Windows version 28.0 (IBM Corp., Armonk, NY).

## Results

A total of 462 patients were screened for inclusion until the sample size of 162 patients was reached. The most common reasons for exclusion were hospital admission from the ED (85 patients), no urinary symptoms documented (44 patients), and presenting with altered mental status. Among the 162 patients included, 103 (64%) had an unplanned healthcare encounter, and of those, 76 (74%) were discharged with inactive empiric therapy. Baseline patient characteristics and demographics are detailed in Table 2. The majority of patients were women (104, 64%) with a median (IQR) age of 59 (33-72) years. The most common UTI classification was complicated lower (71, 44%), followed by complicated upper (57, 35%), catheter-related (24, 15%), and uncomplicated cystitis (10, 6%).

**Table 2:** Baseline and infection characteristics for patients with extended-spectrum β-lactamase (ESBL) urinary tract infection (UTI) treated in an emergency department. IQR: interquartile range

Baseline Characteristics, n (%) or Median (IQR)	Unplanned Encounter (n=103)	No Unplanned Encounter (n=59)	P-value
Age, years	62 (35-75)	48 (29-70)	0.056
Sex, woman	62 (60%)	42 (71%)	0.160
Race, Black/African American	25 (25%)	26 (44%)	0.044
Active health insurance	32 (31%)	27 (46%)	0.096
Charlson Comorbidity Index	3 (0-6)	1 (0-4)	0.085
Diabetes mellitus	40 (39%)	19 (32%)	0.399
Immunocompromised	16 (16%)	6 (10%)	0.338
Chronic kidney disease	16 (16%)	1 (2%)	0.006
Antibiotic use, prior 30 days	31 (30%)	9 (15%)	0.035
ESBL infection, prior six months	17 (17%)	6 (10%)	0.266
Proportion of lower UTI	52 (50%)	29 (49%)	0.870

**Figure 1:**
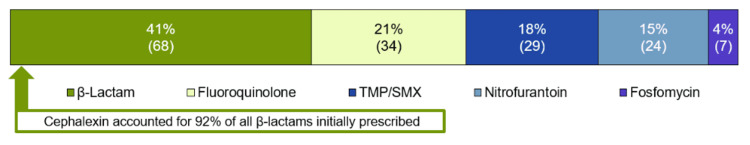
Antibiotic treatment for patients with extended-spectrum β-lactamase urinary tract infection treated in an emergency department. TMP/SMX: trimethoprim/sulfamethoxazole

There were 73 (45%) patients who received at least one dose of IV antibiotics prior to being discharged with oral therapy; 67 (92%) of whom received ceftriaxone, five (7%) received a carbapenem, and one (1%) received a quinolone. Upon discharge, β-lactams were prescribed to 66 (41%) patients. Of these 66 patients, 61 (92%) received cephalexin. Other antibiotic treatment options frequently encountered are detailed in Figure 1. Of the 162 patients, 91 (56%) were initially prescribed an oral antibiotic that was later determined to be ineffective against the culture’s resulting organism. Patients who were prescribed ineffective initial therapy were successfully contacted via telephone in response to their culture results in 67 (74%) of cases, which resulted in a change in therapy in 61 (91%) of those patients. Susceptibility testing revealed that nitrofurantoin demonstrated in vitro activity against 121 (75%) cultured isolates, aminoglycosides in 117 (72%) isolates, trimethoprim/sulfamethoxazole (TMP/SMX) in 66 (41%) isolates, and quinolones in 62 (38%) isolates. In a subgroup analysis of all lower UTI diagnoses, nitrofurantoin was the most effective oral agent, demonstrating in vitro activity against 69 (85%) isolates, followed by TMP/SMX (28, 35%) and quinolones (25, 31%). 

Of the 103 (64%) patients who had an unplanned healthcare encounter, 38 (37%) of them had more than one unplanned healthcare encounter within the 30-day period, for a total of 158 unplanned healthcare encounters. Telephone/virtual encounters were the most common, accounting for 82 (51%) encounters, most of which were prescriber-initiated in response to the resulting culture days following the index encounter. These encounters were followed by ED visits (33, 20%), office/clinic visits (24, 15%), and hospitalizations (19, 12%).

Factors associated with unplanned healthcare encounters are depicted in Table 3. Patients with chronic kidney disease (CKD) (adjusted odds ratio {adjOR}, 3.4; 95% confidence interval {CI}, 1.2-9.5) or those empirically prescribed an oral β-lactam (adjOR, 3.2; 95% CI, 1.5-6.6) were more likely to have an unplanned healthcare encounter. Patients who experienced an unplanned healthcare encounter had a numerically higher comorbidity burden, as measured by the median (IQR) Charlson Comorbidity Index (3 (0-6) versus 1 (0-4); P=0.085). A history of antibiotic use in the preceding 30 days (P=0.035) and a history of ESBL infection in the past six months (P=0.266) were not associated with unplanned healthcare encounters.

**Table 3:** Factors associated with unplanned healthcare encounters in patients with extended-spectrum β-lactamase urinary tract infection treated in an emergency department. unAdjOR, unadjusted odds ratio; adjOR, adjusted odds ratio; CI, confidence interval

Variables	unAdjOR (95% CI)	P-value	adjOR (95% CI)	P-value
Antibiotic use, prior 30 days	2.4 (1.05-5.5)	0.035	2.3 (0.96-5.42)	0.061
Chronic kidney disease	10.7 (1.4-82.6)	0.006	3.37 (1.19-9.49)	0.022
Initial oral β-lactam	3.3 (1.6-6.7)	<0.001	3.16 (1.51-6.60)	0.002

## Discussion

This study found that up to 60% of patients who present to an ED with an ESBL UTI will have at least one UTI-related unplanned healthcare encounter [14]. This is a probable outcome of ineffective initial antibiotic therapy contributing to persistent and worsening infection, which prompts the need for additional healthcare intervention. Forty-one percent of patients received an oral β-lactam on ED discharge, which was found to be associated with a higher likelihood of subsequent unplanned healthcare encounters. Importantly, oral β-lactams were prescribed most frequently for lower UTIs despite nitrofurantoin demonstrating in vitro activity against 85% of ESBL isolates in these cases. These findings underscore the importance of implementing effective antimicrobial stewardship strategies to support optimal empiric antibiotic treatment.

The findings of the present study are congruent with limited published data. Frazee and colleagues found that antibiotic discordance occurred in 46% of ESBL UTI patients seen in an ED [8]. Meaning resulting culture isolates were not susceptible to initial empiric antibiotic treatment in almost half of the patient cases included. In this study of primarily lower UTI, nitrofurantoin demonstrated the highest in vitro activity but was prescribed to only 25% of patients. This is consistent with previously published research describing UTI antibiotic prescribing practices that suggest emergency physicians favor IV and oral β-lactams over nitrofurantoin [15]. The Infectious Diseases Society of America treatment guidelines for UTIs recommend fluoroquinolones, trimethoprim/sulfamethoxazole, oral fosfomycin, and nitrofurantoin as first-line agents, depending on the UTI classification [16]. The article Infectious Diseases Society of America 2023 Guidance on the Treatment of Antimicrobial Resistant Gram-Negative Infections endorses similar recommendations for ESBL cystitis [2]. While ESBL UTIs are often resistant to most oral antibiotics, these data suggest that nitrofurantoin is an underutilized empiric treatment for lower UTI and represents a missed opportunity to improve patient outcomes despite its favorable susceptibility profile for ESBL-producing Enterobacterales. Our group has previously evaluated the utility of oral fosfomycin in ESBL UTIs, supporting its use as a step-down therapy [3].

Hesitancy surrounding nitrofurantoin use may be due to misconceptions related to its use in elderly patients or those with diminished renal function and the potential for ineffectiveness or long-term toxicity. Contemporary data suggest that the previous creatinine clearance labeled cutoff of 60 mL/minute for cystitis indications may be unwarranted, with efficacy studies supporting nitrofurantoin use in patients with creatinine clearances of >30 mL/minute; nitrofurantoin-associated toxicity has only been associated with long-term or prophylactic use [17]. The article American Geriatrics Society 2023 Updated AGS Beers Criteria® for Potentially Inappropriate Medication Use in Older Adults suggests avoiding nitrofurantoin only in patients with a creatinine clearance of <30 mL/minute or in chronic suppression indications [18]. An alternative hypothesis could be related to UTI diagnostic challenges and limited infection data at the time of ED care. As the emergency physician does not have timely access to phenotypic microbiology results for urine culture testing at the time the antibiotic prescription is written, empiric antibiotic selection to cover an ESBL-producing organism may not be considered. However, antibiogram data and national guidelines still position nitrofurantoin as a preferred therapy in these scenarios. Additionally, the clinician may not have the appropriate data (patient history or imaging) necessary to distinguish between lower or upper UTI, whereas nitrofurantoin should not be used in upper UTI due to poor target site penetration [4,17]. While this study highlights the underutilization of nitrofurantoin despite favorable susceptibility patterns, it was not designed to explore clinician knowledge, perceptions, or institutional barriers that may influence prescribing behavior. Future prospective studies should evaluate the role of decision support tools and stewardship interventions in improving empiric antibiotic selection and clinical outcomes in patients with ESBL UTIs.

Limitations

While our study provides valuable insights, it is important to acknowledge several limitations. This study was conducted within a single health system and may restrict the generalizability of our findings beyond our community and antibiogram. However, this study’s ED catchment area is representative of an extremely diverse patient population. When assessing adequate antimicrobial therapy, only in vitro activity was taken into consideration as antibiotic dosing was not captured. Previous data have shown suboptimal dosing in the study population; therefore, inadequate dosing could have contributed to the unplanned healthcare encounters captured [4]. However, all participating EDs have access to standardized UTI-specific order panels and local antibiograms designed to support appropriate empiric antibiotic selection and dosing to mitigate this risk. The retrospective nature of the study is also a limitation. Due to the retrospective design, medication adherence and adverse drug reactions were not captured. These missing data could both underestimate the true burden of treatment failure and overestimate the appropriateness of empiric antibiotic selection. While there is a possibility of data abstract bias, a single author (JMB) facilitated data abstraction using a standardized data collection form. Additionally, the follow-up of urine culture results post discharge is handled differently across study sites and is dependent on local workflows. While each site ensures review of culture and susceptibility data, the process varies, ranging from follow-up by ED-based advanced practice providers to registered nurses or clinical pharmacists. In cases where empiric therapy is deemed inappropriate based on final susceptibilities, follow-up contact is initiated to adjust treatment. This variability in follow-up practices represents a limitation of the study, as it may have influenced the rate or timing of unplanned healthcare encounters resulting from culture callbacks.

## Conclusions

This study suggests that patients with ESBL UTIs are at an increased risk for unplanned healthcare encounters. The majority of patients received inadequate antibiotic therapy that facilitated the unplanned healthcare encounter. Education and/or leveraging the electronic health record to support the use of first-line therapies for lower UTIs, such as nitrofurantoin when appropriate, represents future directions of this research. Additionally, integrating forecasting models to support point-of-care decision-making offers a valuable antimicrobial stewardship strategy to improve outcomes in a challenging patient population and care setting.

## Appendices

Table 4 shows the reason for patient exclusion.

**Table 4:** Reason for patient exclusion SCr, serum creatinine; CFU, colony-forming unit; UTI, urinary tract infection; CrCL, creatinine clearance

Reason for Exclusion	Number of Patients (n=300)
Admitted to hospital	85
Asymptomatic	44
Did not receive treatment	33
Altered mental status	26
No SCr on file	24
CFU count below threshold	23
Age of <18 years old	21
Polymicrobial UTI	19
CrCL of <30 mL/minute	9
On antibiotics prior to encounter	7
Renal transplant recipient	3
Alternative diagnosis	3
Discharged with parenteral antibiotics	2
Urological anatomy abnormality	1
